# Astaxanthin Modulation of Signaling Pathways That Regulate Autophagy

**DOI:** 10.3390/md17100546

**Published:** 2019-09-23

**Authors:** Suhn Hyung Kim, Hyeyoung Kim

**Affiliations:** Department of Food and Nutrition, Brain Korea 21 PLUS Project, College of Human Ecology, Yonsei University, Seoul 03722, Korea; cigdoli2@naver.com

**Keywords:** AMP activated protein kinase (AMPK), autophagy, astaxanthin, cellular homolog of murine thymoma virus akt8 oncogene (Akt), mitogen-activated protein kinases (MAPK), c-Jun N-terminal kinase (JNK), p38

## Abstract

Autophagy is a lysosomal pathway that degrades and recycles unused or dysfunctional cell components as well as toxic cytosolic materials. Basal autophagy favors cell survival. However, the aberrant regulation of autophagy can promote pathological conditions. The autophagy pathway is regulated by several cell-stress and cell-survival signaling pathways that can be targeted for the purpose of disease control. In experimental models of disease, the carotenoid astaxanthin has been shown to modulate autophagy by regulating signaling pathways, including the AMP-activated protein kinase (AMPK), cellular homolog of murine thymoma virus akt8 oncogene (Akt), and mitogen-activated protein kinase (MAPK), such as c-Jun N-terminal kinase (JNK) and p38. Astaxanthin is a promising therapeutic agent for the treatment of a wide variety of diseases by regulating autophagy.

## 1. The Autophagy Machinery

Autophagy, which literally means ‘to eat oneself’, is a catabolic process that degrades and recycles various cellular constituents, including dysfunctional organelles. Christian de Duve coined the name autophagy for the lysosomal-mediated pathway first described by Ashford and Porter [1,2]. More recently, three types of autophagy have been identified, including macroautophagy, microautophagy, and chaperone-mediated autophagy. The most thoroughly studied type is macroautophagy (hereafter referred to as autophagy) that involves the sequestration and recycling of expendable cytoplasmic constituents, such as damaged organelles and unused proteins. In this process, cytosolic components are first sequestered into a cup-shaped double-membrane structure, known as a phagophore, which elongates and matures into the double-membrane vesicle known as an autophagosome. The autophagosome is trafficked to the lysosome with which it fuses to form an autolysosome. The contents of the autolysosome that formed in this manner are degraded through the actions of lysosomal and vacuolar hydrolases. The reusable macromolecules are transported back to the cytosol through the actions of membrane permeases [3].

In the course of understanding the precise mechanism of autophagy, several genes that are essential to the induction of autophagy have been discovered. Autophagy-related genes (ATG) are essential for autophagosome formation. These genes encode the core molecular machinery of autophagy, and are classified into four major subgroups [4]. The first group encodes the unc-51-like kinase (ULK) complex, composed of ULK1/2 (mammalian homolog of yeast Atg1), mAtg13 (mammalian homolog of yeast Atg13), and FIP200 (mammalian homolog of yeast Atg17). The ULK complex is the initial regulator of the autophagy pathway that recruits proteins to the phagophore assembly site (PAS) and initiates the organization of the phagophore membrane [5]. The ULK complex acts downstream of the signaling complex, which is the known mammalian target of the rapamycin (mTOR) complex 1 (mTORC1) [6]. Under nutrient-rich conditions, mTOR-signaling results in mAtg13 phosphorylation, thereby facilitating the dissociation of the ULK1/2-mAtg13-FIP200 complex. However, under nutrient-starvation conditions, mTORC1 is inactivated, and dephosphorylated ULK1/2 and mAtg13 form a stable complex that activates the autophagy signal [6,7].

The second group is transmembrane carrier proteins that are required for membrane organization of the autophagosome. The mammalian homolog of yeast Atg9 (mAtg9) is the major transport protein, which alternates between the PAS and peripheral donor sites, such as the trans-Golgi network and the endosomes [8]. Upon the induction of autophagy, mAtg9 transiently co-localizes with the PAS and transports membrane lipids from donor organelles to the phagophore, before returning to the peripheral sites around the trans-Golgi network where mAtg9 normally localizes [8,9]. Another transmembrane carrier protein, the vacuole membrane protein 1 (VMP1), induces the formation of ultrastructural features of autophagy and auophagosome formation [10].

The third component of the core molecular machinery of autophagy consists of two ubiquitin-like (Ubl) protein conjugation systems, both of which play fundamental roles in regulating the elongation and expansion of the phagophore [11]. The first system consists of mAtg12 (mammalian homolog of Atg12) and its conjugate mAtg5 (mammalian homolog of Atg5). The other system, LC3 (mammalian homolog of Atg8), is processed into an active form upon the induction of autophagy. LC3 is first cleaved to generate the cytosolic form LC3-I, and it then is conjugated to phophatidylethanolamine (PE) to generate the lipidated form LC3-II. LC3-II localizes to the outer and inner membrane of the autophagosome [12]. The mAtg12-mAgt5 and LC3 systems work in concert to determine the size of autophagosomes and both systems are essential for autophagosome closure [13,14].

The class III phosphatidylinositol 3-kinase (PI3K) complex is the fourth component of the autophagy machinery. Through its membrane association and kinase activity, class III PI3K regulates vesicular trafficking and vacuolar protein sorting (Vps) [15,16,17,18]. The class III PI3K hVps34 (human homolog of yeast Vps34) works in concert with its regulatory adaptor proteins p150 (mammalian homolog of Vps15) and Beclin 1 (mammalian homolog of Atg6 (also known as Vps30)). The protein kinase p150 is required for the association of hVps34 with the phagophore membrane from which it exerts its lipid kinase activity [19]. Beclin 1 binds to other regulatory proteins, such as Atg14L (mammalian homolog of Atg14), UVRAG (mammalian homolog of Vps38), and Rubicon [20]. Atg14L directs the class III PI3K complex to the phagophore, where it initiates both the recruitment of the ATG machinery and phagophore nucleation [21,22,23]. Whereas, Atg14L plays a role in the early vesicle formation at the PAS, UVRAG serves in autophagosome maturation and accelerates the delivery and degradation of autophagic cargo [23,24,25,26]. Rubicon negatively regulates autophagosome formation [26].

A basal level of autophagy is essential for cellular homeostasis and survival, because it eliminates damaged organelles that may become toxic and it leads to the turnover of cellular materials, such as long-lived proteins [27]. Increased levels of autophagy constitute a protective response to certain diseased states as well to other sources of stress, such as nutrient starvation, oxidative stress, radiation, and hypoxia [28,29].

## 2. Signaling Pathways That Regulate Autophagy

Recent studies have identified several signal transduction pathways that regulate autophagy in response to various environmental conditions. The autophagy pathway has been elucidated in relation to the mTOR-signaling pathway because autophagy was initially studied in the context of cellular responses to nutrient deprivation. mTOR is the direct on-and-off switch of the autophagy pathway in response to growth factors, nutrient signals, and cellular energy status [30,31]. mTOR signaling directly inhibits autophagy through phosphorylation of the proteins that comprise the ULK complex [32]. Table 1 provides a list of the signaling pathways that regulate autophagy and are described below.

### 2.1. AMP-Activated Protein Kinase (AMPK)

One of the direct upstream regulators of the mTOR pathway, which acts in response to nutrients and cellular energy status, is AMP-activated protein kinase (AMPK). Kimura et al. reported that activation of AMPK inhibits p70 S6 kinase (p70 S6K) of the mTOR pathway, and that glucose or amino acid withdrawal activates AMPK and inhibits mTOR signaling [33]. In particular, upon cellular energy starvation, AMPK directly phosphorylates and thereby activates the mTOR inhibitor tuberous sclerosis complex 2 (TSC2). AMPK-activated TSC2 inhibits the mTOR pathway that involves cell growth and survival [34]. Oxygen deprivation was observed to promote LC3 processing and autophagosome formation in tumor cells, which are mediated by AMPK activation and by downregulation of mTOR [35]. Tumor necrosis factor (TNF)-related apoptosis-inducing ligand (TRAIL) and resveratrol were observed to stimulate autophagic cell death in breast epithelial cells and chronic myelogenous leukemia cells, respectively, via AMPK activation and the suppression of downstream mTOR signaling [36,37]. AMPK also mediated autophagy triggered by an increase in the level of free cytosolic Ca^2+^ [38].

In addition, AMPK also directly interacts with ULK1 by binding to its proline-rich region domain. AMPK binding to ULK1 is required for autophagy induction, and treatment with an AMPK activator inhibits mTORC1 and stimulates autophagy [39]. AMPK directly phosphorylates ULK1 at Ser 555, which is required for the induction of autophagy in response to the nutrient status [40]. Kim et al. reported differential phosphorylation events on ULK1 mediated by AMPK and mTOR. Specifically, under glucose starvation, AMPK was observed to promote autophagy by directly activating ULK1 through the phosphorylation of Ser 317 and Ser 777, whereas under nutrient sufficiency, high mTOR activity was found to prevent ULK1 activation by phosphorylating ULK1 Ser 757 and thus preventing the interaction between ULK1 and AMPK [41].

### 2.2. Phosphatidylinositol 3-Kinase (PI3K)/Cellular Homolog of Murine Thymoma Virus akt8 Oncogene (Akt)

The cellular homolog of murine thymoma virus akt8 oncogene (Akt) is another signaling kinase that can regulate autophagy through the modulation of the mTOR pathway. Unlike AMPK, PI3K/Akt signaling activates the mTOR pathway, and thus inhibits autophagy [42]. Silencing the Akt gene or administrating PI3K and Akt inhibitors increases autophagy [43]. Akt can modulate the activation of mTORC1 by acting on the mTORC1 negative regulator TSC2. Activated Akt (p-Akt) phosphorylates TSC2 and thereby blocks the inhibition of mTORC1 signaling [44]. Activation of the PI3K/Akt/mTOR signaling was observed to significantly suppress starvation-induced autophagy [45,46]. Furthermore, signaling molecules, such as IGF-1 and IL-13, reportedly inhibit autophagy via activation of the Akt-signaling pathway [47,48]. On the other hand, down-regulation of active p-PI3K, p-Akt, and p-mTOR by ceramide, resveratrol, brain-derived neurotrophic factor (BDNF), or oleanolic acid stimulates protective autophagy in pathological disease models, such as myocardial hypertrophy, ischemic brain injury, or diabetic nephropathy [49,50,51,52].

The aberrant activation of the PI3K/Akt signaling pathway is commonly observed in cancer cells and thus represents a potential target for the development of anticancer therapeutics [53]. Indeed, the inhibition of the PI3K/Akt pathway in various cancer cell lines using the natural products matrine and paeonol significantly decreased cancer cell viability by promoting autophagy [54,55].

Akt can also regulate autophagy through other mechanisms, although the regulation of autophagy by Akt usually occurs via the modulation of the mTOR pathway. For instance, Wang et al. reported that Akt-mediated regulation of autophagy and tumorigenesis occurs through Beclin 1 phosphorylation [56]. Akt phosphorylates Beclin 1 at S295 and S234, which in turn blocks the interaction between Beclin 1 and class III PI3K. Akt inhibition or the expression of Akt phosphorylation-resistant Beclin 1 serves to increase autophagy and alleviate tumorigenesis [56].

Akt also regulates autophagy in response to reactive oxygen species (ROS). Particle matters derived from cooking oil fumes increased ROS production and autophagy in human umbilical vein endothelial cells (HUVEC), as a result of decreased levels of p-PI3K, p-Akt, and p-mTOR [57]. The antioxidant N-acetlycysteine (NAC) was observed to reverse these effects, which shows that ROS production stimulates Akt/mTOR inhibition-mediated autophagy [57]. The exposure of mouse osteoblastic MC3T3-E1 cells to high levels of glucose also induced autophagy by decreasing the levels of p-Akt and p-mTOR [58]. NAC treatment restored Akt/mTOR activation and reduced autophagic cell death, which demonstrates the role of the ROS-Akt-mTOR signaling axis in the regulation of autophagy [58].

### 2.3. c-Jun N-Terminal Kinase (JNK)

Mitogen-activated protein kinases (MAPKs) are activated in response to various stressors. MAPK signaling can modulate autophagy, ultimately regulating the cellular response under stress conditions. The MAPK c-Jun N-terminal kinase (JNK) is the major stress-activated protein kinase that modulates cellular signaling through the phosphorylation of its downstream target c-jun. Here, we list the major experimental findings that have defined the role of the JNK-c-jun pathway in autophagy induction. (1) The neurotoxin N-methyl-d-aspartate (NMDA) induces autophagic neuronal cell death by activating JNK [59]. (2) z-VAD induces autophagy by modulation of Atg7 and Beclin 1, and oncogenic H-ras induces autophagy by increasing Atg5 expression, which are both mediated by the activation of the JNK pathway [60,61]. (3) The presence of a JNK chemical inhibitor or the deletion of the genes encoding JNK1 and JNK2 inhibit the autophagy of activated CD4+ T cells, which indicates that the induction of autophagy is mediated by JNK [62]. (4) Ceramide promotes autophagy in human nasopharyngeal carcinoma cells and hepatocarcinoma cells through the activation of JNK and its downstream target c-jun, which ultimately increases Beclin 1 mRNA expression. Ceramide treatment also induces autophagy in neural progenitor cells through JNK activation. Activation of JNK and the subsequent phosphorylation of c-jun promotes the transcription of the LC3 gene by binding to its promoter region. Blocking JNK or c-jun activity abrogates ceramide-induced autophagy [63,64]. (5) The ectopic expression of ubiquitin-binding histone deacetylase 6 (HDAC6) in liver cancer cells promotes JNK-mediated Beclin 1 expression and LC3II conversion [65].

JNK activation is thought to be a key regulator in ROS-mediated autophagic cell death [66]. Bufalin stimulates JNK2 activation by elevating the ROS levels, and activates JNK2-induced autophagy by up-regulation of Beclin 1 and ATG5 [67]. Wu et al. reported that JNK is not involved in starvation-induced autophagy, but rather it induces autophagy in response to oxidative stress. The onset of oxidative stress in the intestine is known to activate JNK and induce protective autophagy. Activated JNK increases the expression of ATG genes, which in turn renders cells more resistant to oxidant-induced cytotoxicity [46].

Recently, it has been discovered that c-jun can regulate the expression of Bcl-2 and Bax. Bcl-2 directly interacts with the BH3 domain of Beclin 1, and thereby prevents Beclin 1 from forming a complex with hVps34 and PI3K [68]. Under normal conditions, Bcl-2 is associated with Beclin 1, and this association prevents Beclin 1 from inducing autophagy [69]. Wei et al. reported that nutrient starvation activates JNK1, and hence, JNK1-catalyzed multi-phosphorylation of Bcl-2 results in the dissociation of the Bcl-2-Beclin 1 complex and, thus, the induction of autophagy [70]. Notably, both starvation conditions and ceramide treatment can induce autophagy via the JNK-1-catalyzed phosphorylation of Bcl-2 [70,71].

Anti-TRAIL receptor 2(TR2) single-chain fragment variable, HW1, is known to induce autophagic cell death in cancer cells via JNK activation. HW1 activates both JNK and p38, but only the JNK-specific inhibitor blocks HW1-induced autophagy. The study indicates that JNK activation is responsible for the autophagy induction by HW1 [72]. It has been shown that the HW1-stimulated activation of JNK increases Beclin 1 expression and induces p53 activation and mTOR inhibition [73]. The HW1-induced increase of Beclin 1 expression was found to result from direct phosphorylation of Bcl-2 [73].

### 2.4. p38

p38 is another MAPK that is activated by environmental and genotoxic stressors. The nature of the stressor determines the regulatory effect of p38 on the autophagic pathway. Under inflammatory conditions, p38 negatively regulates autophagy. The inflammatory agent lipopolysaccharide (LPS) has been shown to inhibit autophagy and promote inflammation in microglial cells via the phosphorylative activation of p38 [74]. Activated p38 directly phosphorylates ULK1 and thereby blocks ULK1 activation of its downstream effector ATG13 [74]. Notably, it has been reported that TNF-α induces autophagy by decreasing the expression of p-p38 and that the inhibition of p38 activation by an exogenous chemical inhibitor enhances TNF-α-induced autophagy [75].

The mechanism by which p38-mediates the autophagic pathway in cancer cells has been extensively studied. The anti-tumor chemical E Platinum induces autophagy in various carcinoma cell lines by decreasing phosphorylation of p38 and inhibiting mTOR activation [76]. Triterpenes from *Ganoderma Lucidum* were found to induce autophagy in human colon cancer HT-29 cells in vitro, as well as in HT-29 xenograft tumors in vivo, by suppressing p38 activation [77]. Come et al. reported that p38α kinase modulates autophagy in a cell-specific manner [78]. Specifically, the suppression of p38α kinase activation notably increases autophagy in colorectal cancer cells, including HT-29 cells, but not in other carcinoma cell types [78]. p38 activation has been shown to be a key determinant in the response of colon cancer HCT-116 cells to treatment with 5’-fluorouracil by controlling the balance between apoptosis and autophagy [79]. The inhibition of p38 activation was observed to promote autophagic cell death [79]. On the other hand, the overexpression of constitutively active MAPK14/p38α kinase in colon carcinoma cells markedly increases survival autophagy, thereby making the cells more resistant to the toxicity of irinotecan [80]. Likewise, the diterpenoid oridonin induces autophagy in human cervical carcinoma HeLa cells, and increases p38 gene expression and p38 phosphorylation [81].

Oxidative stress also creates a link between p38 and autophagy. LPS-induced oxidative stress and p38 activation in skeletal muscle results in the increased expression of Atg7 and Beclin 1 [82]. Furthermore, the administration of a p38 chemical inhibitor was found to block H_2_O_2_-induced expression of Atg genes such as Atg 7 and Beclin 1 [82]. Together, these findings demonstrate that oxidative stress induces autophagic gene expression via the activation of p38 [82]. Resveratrol treatment protects embryonic rat heart-derived cells subjected to H_2_O_2_-induced oxidative stress by activating p38 and increasing autophagy [83]. ER stress also stimulates autophagy by increasing p38 phosphorylation. [84].

p38 regulates the autophagic pathway through the direct modulation of ATG genes. The signaling complex formed between growth arrest and DNA-damage-inducible, beta (Gadd45β) and the MAP kinase kinase kinase (MEKK) 4, activates p38 by phosphorylation and results in the localization of p-p38 to autophagosomes. Gadd45β/MEKK4-activated p38 inhibits starvation-induced autophagy by phosphorylating Atg5 and blocking autophagosome fusion with lysosomes [85]. Webber et al. reported that p38 negatively regulates the interaction between p38-interacting protein (p38IP) and mAtg9, thereby alerting the trafficking of mAtg9 and autophagosome formation [86]. Selenite-induced stress resulted in the upregulation of autophagic proteins, such as Beclin 1, Lamp-1, and cathepsin D, and increased degradation of p62, each occurring through p38 activation [87].

## 3. The Effect of Astaxanthin on the Signaling Pathways That Regulate Autophagy

### 3.1. Astaxnathin and AMPK Signaling

The effect of the antioxidant astaxanthin on AMPK signaling has not been extensively studied. However, the findings from the few studies that have been reported indicate that astaxanthin acts as a positive AMPK regulator. Specifically, astaxanthin was observed to inhibit lipogenesis and fat accumulation in the liver and to inhibit hepatic apoptosis in oleic acid-induced hepatic steatosis [88]. Astaxanthin’s anti-steatotic properties are attributed to its ability to activate AMPK signaling, as reflected by the observed increase in the ratio of phosphorylated vs unphosphorylated AMPK (p-AMPK/AMPK). Astaxanthin-induced AMPK activation was found to negatively regulate lipogenesis and promote fatty acid oxidation [88]. Furthermore, it has been shown that the intake of esterified astaxanthin that was extracted from *Haematococcus pluvialis* increases the running time of mice to exhaustion as a result of the increased level of total AMPK in the skeletal muscle [89]. The fact that the activation of AMPK promotes autophagy via mTOR inhibition and direct activation of the ULK1 complex suggests that astaxanthin may modulate the induction of autophagy.

### 3.2. Astaxanthin and PI3K/Akt Signaling

The regulatory effect of astaxanthin on PI3K/Akt has been extensively investigated. Astaxanthin exhibits protective effects against cytotoxicity and apoptotic cell death in H_2_O_2_-stimulated mouse neural progenitor cells, acetaldehyde-stimulated SH-SY5Y cells, glutamate-stimulated hippocampal HT22 cells, and homocysteine-stimulated rat hippocampal neurons [90,91,92,93]. Astaxanthin was observed to increase the level of active p-Akt in human retinal pigment epithelial cells, which in turn results in decreased apoptotic cell death and increased levels of Nrf-2-induced antioxidant enzymes. Notably, treatment of the cells with an inhibitor of Akt phosphorylation blocked these responses [94].

The protective property of astaxanthin against neurodegeneration is well established. Astaxanthin treatment was found to reduce apoptotic cell death and neuronal damage in vitro and in vivo by up-regulating p-PI3K and p-Akt [95,96,97]. Astaxanthin attenuates spinal cord injury-induced neuropathic pain and the death of motor neurons, by restoring p-Akt to a normal level [98]. Through the activation of PI3K and Akt, astaxanthin also increases the proliferation of neural progenitor cells and neural stem cells, and improves their cell potency [99,100]. In addition, astaxanthin is known to increase the phosphorylation of Akt and mTOR in the Kupffer cells of the hypoxia and reoxygenation-induced mice model of ischemia-reperfusion injury (IRI) [101].

Astaxanthin improves cell survival under oxidative inflammation conditions via the activation of Akt signaling. In particular, astaxanthin was observed to stimulate the expression of PI3K, Akt, and p-Akt in diabetic rats, and in this manner to down-regulate pro-apoptotic proteins and reduces the cognitive deficits that are caused by inflammation [102,103]. In addition, stimulation of Akt/Bad signaling by astaxanthin showed beneficial effects in post-injury models, wherein the levels of p-Akt and p-Bad are elevated after burn insult and subarachnoid hemorrhage. Astaxanthin treatment was found to further increase the level of p-Akt, which results in a decrease in apoptotic cell death and inflammatory-induced damage. Importantly, the presence of an Akt inhibitor was shown to abolish the protective effect of astaxanthin [104,105,106]. Astaxanthin exhibits anti-proliferative properties in cancer cells through negative regulation of Akt signaling. Astaxanthin has been observed to inhibit cell proliferation and induce apoptosis in several types of carcinoma cells, by decreasing the p-Akt/Akt ratio, thereby down-regulating Akt downstream signaling pathways, such as those of NF-κB, Wnt, and STAT3. In human hepatocellular carcinoma cells and in DMBA-induced oral cancer in hamsters, astaxanthin treatment was shown to inhibit Akt kinase activation and NF-κB and β-catenin/Wnt signaling, which resulted in the inhibition of cell proliferation and the stimulation of apoptosis [107,108]. In addition, astaxanthin induces apoptosis and reduces cancer hallmarks in both in vitro and in vivo models of oral squamous cell carcinoma via the inhibition of PI3K and p-Akt, and their downstream targets NF-κB and STAT3 [109]. Astaxanthin is also known to also reduce the viability of non-small cell lung cancer cells and enhance the cytotoxicity of mitomycin C through down-regulation of p-Akt, and hence Akt-mediated the down-regulation of the carcinogenic factor Rad51 [110].

Regarding autophagy, astaxanthin has been shown to stimulate the hepatic autophagy pathway operative in an experimental model of non-alcoholic fatty liver disease (NAFLD) [111]. Hepatic autophagy breaks down stored lipid droplets in the liver, which can prevent, or reduce the severity of hepatic steatosis. Astaxanthin treatment reduces hepatic lipid accumulations and alleviates hepatic inflammation in high-fat-fed C57BL/6J mice [111]. Astaxanthin also induces hepatic autophagy, as indicated by the observed increase in the autophagy marker LC3II and the up-regulation of the key regulators of autophagy, such as LAMP1, LAMP2, ATG7, and Beclin 1. Astaxanthin treatment reportedly inhibits the activation of Akt, leading to an induction of autophagy via the Akt/mTOR pathway. In addition, astaxanthin was observed to simulate PPAR-α and inhibit PPAR-γ, and thus astaxanthin might also affect the hepatic autophagy pathway via the direct regulation of PPAR [111].

Zhang et al. reported that astaxanthin alleviates cerulein-induced acute pancreatitis not only by reducing the inflammatory response, but also by inhibiting pancreatic cell apoptosis and autophagy [112]. Specifically, pretreatment of mice with astaxanthin before cerulein injection, reduced the expression of inflammatory cytokine genes at the mRNA and protein levels and alleviated the pancreatic histophathological abnormalities. Furthermore, astaxanthin was observed to inhibit the expression of pro-apoptotic proteins and pro-autophagic proteins. In particular, cerulein up-regulates the expression of the LC3 and Beclin 1 genes at the mRNA and protein level, whereas astaxanthin pretreatment down-regulates LC3 and Beclin 1 and increases the expression of p62 and Bcl-2. In addition, astaxanthin decreases JAK and STAT3 mRNA expression, as well as the levels of JAK1, JAK2, and p-STAT3. These results suggest that astaxanthin can inhibit inflammation and autophagy in the pancreas by inhibiting the JAK/STAT3 pathway [112].

Astaxanthin exerts its protective effect on liver fibrosis by suppressing pro-fibrogenic factors and autophagy in hepatic stellate cells (HSCs) [113]. In particular, astaxanthin decreases the levels of LC3 and Beclin 1 (mRNA and protein expression) in HSCs. Notably, fewer lysosomes and autophagosomes were observed in the astaxanthin-treated mice when compared to the CCL4-stimulated mice. Astaxanthin was also shown to down-regulate autophagy markers in hepatic HSC-T6 cells. Given that the cytokine TGF-β1 is the stimulator of HSCs in liver fibrosis, and that autophagy can further stimulate HSC activation through degradation of lipid droplets to provide energy, it is reasonable to conclude that astaxanthin ameliorates HSC activation-induced liver fibrosis by suppressing TGF-β1 and the downstream autophagic pathway [113].

### 3.3. Astaxanthin and JNK Signaling

The therapeutic properties of astaxanthin are likely to result from its impact on MAPKs in view of the fact that MAPK pathways govern the majority of the cellular responses to various stimulants and stressors. In fact, the cytoprotective effects of astaxanthin under stress-induced pathological conditions are often mediated by the inhibition of the JNK pathway. Astaxanthin has been shown to significantly reduce JNK1 and JNK2 activation in HLE cells exposed to UVB radiation [114], as well as to suppress JNK phosphorylation in cells activated by stimulants, such as TNF-α, cobalt, dextran sulfate sodium, and insulin [115,116,117]. Furthermore, astaxanthin relieves the inflammatory response and protects cell viability by inhibiting LPS-induced, and palmitate-induced, phosphorylation of JNK [118,119]. Zhang et al. reported that astaxanthin decreases the level of JNK and p-JNK and, in this manner, prevents oxidative stress, inflammation, and histopathological liver damage in the acetaminophen-stimulated liver injury mouse model [120]. In addition, it has been shown that a high fat high fructose diet (HFFD) feed to mice or alternatively, palmitate treatment of MIN6 β-cells in vitro, cause inflammatory ER stress through elevation of JNK activation, and that administration of astaxanthin inhibits this activation [121,122].

Astaxanthin mitigates fluctuating high glucose-induced inflammatory stress and apoptosis through inhibition of JNK phosphorylation [123]. On the other hand, the administration of a *Hematococcus pluvialis* extract containing astaxanthin was found to suppress cell growth and induce apoptosis in human colon cancer HCT-116 cells by increasing the level of p-JNK [124]. Taken together, these findings suggest that astaxanthin can inhibit or facilitate JNK signaling, depending on the system.

In the study on autophagy, astaxanthin protects hepatocytes from ConA-induced autoimmune hepatitis by inhibiting apoptosis and autophagy, and by blocking JNK-mediated increases in pro-apoptotic proteins and pro-autophagic proteins [125]. Specifically, whereas ConA induces the activation of inflammatory mediators, such as TNF-α, IL-6, IL-1β, and p65, and it triggers histopathological inflammatory damage, astaxanthin reverses these effects. Astaxanthin was also observed to reduce the levels of pro-appototic proteins Bax and caspase-9, as well as the autophagy-related proteins Beclin 1 and LC3. In addition, astaxanthin was shown to inhibit TNF-α-induced TRAF2 activation and the phosphorylation of JNK, thereby blocking the phosphorylation of Bcl-2, and hence the release of Beclin 1 from the Bcl-2/Beclin 1 complex [125].

### 3.4. Astaxanthin and p38 Signaling

Some studies have shown that astaxanthin activates p38. In particular, the astaxanthin-rich *Hematococcus pluvialis* extract is reported to inhibit cell growth and induce apoptosis in human colon cancer cells by stimulating the phosphorylation of p38 [124]. Moreover, p38 activation reportedly decreases the expression of DNA damage recognizing protein and increases cytotoxicity in human lung carcinoma cells [126].

Other studies suggest that astaxanthin inhibits p38 activity. The pertinent findings from these studies are as follows. (1) It has been shown that astaxanthin treatment suppresses stimulant-activated p38 [114,116,118,120] and prevents H_2_O_2_-induced apoptotic cell death in mouse neural progenitor cells through the inhibition of p38 [90]. (2) Abdelzaher et al. reported that astaxanthin blocks the increase in p38 phosphorylation and prevents inflammation and apoptosis in HUVEC cells received glucose-induced oxidative stress [123]. (3) Numerous studies that were carried out with human neuroblastoma SH-SY5Y cells exposed to a variety of pathological stimulants have demonstrated that, by blocking p38 activation, astaxanthin suppresses apoptosis and ameliorates oxidative stress, ER stress, and mitochondrial dysfunction [91,127,128,129]. (4) Other studies have shown that astaxanthin inhibits the β-amyloid-induced apoptosis of PC12 cells [130], and preserves cell viability in palmitate-simulated mesenchymal stem cells [119]. (5) Yang et al. reported that astaxanthin binds to the p38 active site thereby inhibiting p38 phosphorylation and hence, the cognitive defects and neuronal damage in environmental tobacco smoke (ETS)-exposed mice [131]. (6) Astaxanthin was observed to mitigate cyclophosphamide-stimulated oxidative cellular damage and early hepatocarcinogenesis by blocking p38 phosphorylation by cyclophosphamide [132]. (7) The results that were reported by Sakai et al. indicate that astaxanthin treatment can prevent colitis in mice by inhibiting dextran sodium sulfate (DSS)-induced p38 phosphorylation and that, in human colonic epithelial cells, astaxanthin inhibits TNF-α-stimulated phosphorylation of p38 [115]. (8) Astaxanthin inhibition of p38 phosphorylation restores motor function following spinal cord injury [133] and reduces IL-1β-induced expression of matrix metalloproteinase in chondrocytes [134].

In the studies on autophagy, Li et al. reported that astaxanthin can alleviate functional and structural abnormalities in the liver, reduce the expression of inflammatory cytokines, and suppress hepatocyte apoptosis and autophagy in a hepatic IRI model [135]. Analysis of the major autophagy-related proteins revealed that the astaxanthin treatment decreased the levels of Beclin 1 and LC3 and decreased the level of p62. Astaxanthin also inhibited IRI-induced phosphorylation of MAPKs, p38, extracellular-signal-regulated kinase (ERK), and JNK, and normalized the levels of their downstream effectors, such as Bax and Bcl-2. Taken together, these results suggest that astaxanthin reduces autophagy by modulating MAPK-mediated Bax and Bcl-2 activation and the subsequent release of Beclin 1 [135].

## 4. Conclusions

**Figure 1:**
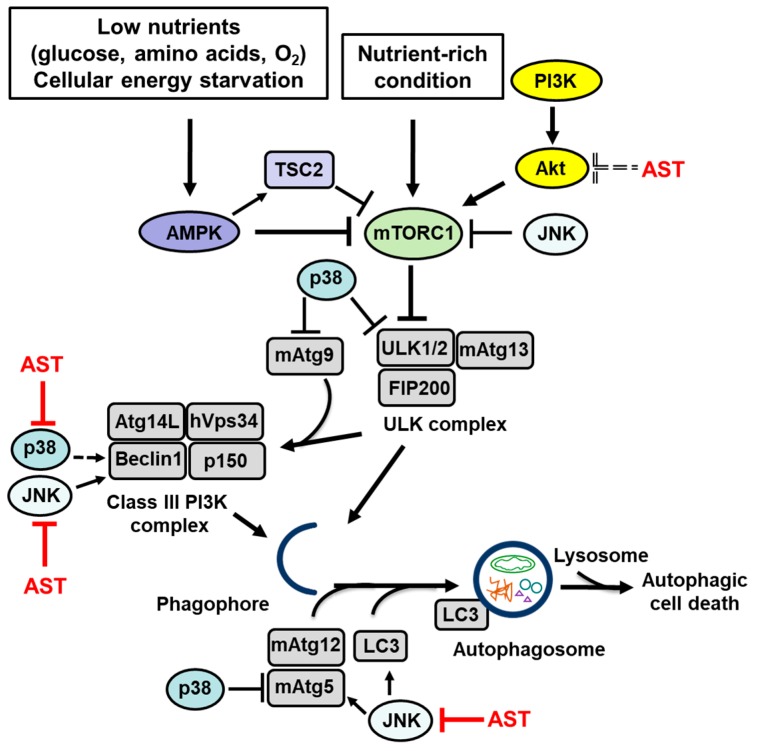
The signaling pathways that regulate autophagy and autophagy-related proteins that are affected by astaxanthin (AST). Low nutrients levels (glucose or amino acid withdrawal, oxygen deprivation) and cellular energy starvation activate AMPK, which inactivates mTORC1 via stimulation of TSC2. When mTORC1 is inactivated, dephosphorylated ULK1/2 and mAtg13 form a stable ULK complex (ULK1/2-mAtg13-FIP200 complex). The ULK complex is the regulator of the autophagy pathway that initiates organization of the phagophore membranes. Upon induction of autophagy, mAtg9, a major transport protein, transports membrane lipids from donor organelles to the phagophore. Two ubiquitin-like protein conjugation systems, mAtg12 along with its conjugate mAtg5 and LC3 play roles in elongation and expansion of the phagophore to induce autophagosome formation and closure. In the Class III PI3K complex, hVsp34 works in concert with its regulatory adaptor proteins p150 and Beclin 1 to regulate vesicular trafficking and vacuolar protein sorting. p150 is required for association of hVsp34 with the phagophore membrane. Binding of Beclin 1 to Atg14L directs the class III PI3K complex to the phagophore, where it initiates both recruitment of the Atg machinery and phagophore nucleation. Under nutrient-rich conditions, mTOR signaling is activated. Activated mTORC1 induces phosphorylation of mAtg13 and dissociation of the ULK complex leading to inhibition of autophagy. PIK3/Akt activates the mTOR pathway leading to inhibition of autophagy. JNK inhibits the mTOR pathway and increases expression of Beclin 1, mAtg5, and LC3, resulting in induction of autophagy. p38 increases expression of Beclin 1 and induces autophagy. In contrast, p38 phosphorylates ULK1 and blocks ULK1 activation of its downstream effector mAtg13. Also, p38 inhibits starvation-induced autophagy by phosphorylating mAtg5 and blocking autophagosome fusion with lysosomes. p38 alters trafficking of mAtg9 and autophagosome formation. In the context of autophagy, AST stimulates or inhibits autophagy by increasing or decreasing LC3 and Beclin 1 depending on the experimental model used. In relation to Akt signaling, AST inhibits the activation of Akt, leading to induction of autophagy via the Akt/mTOR pathway and increases expression of Beclin 1 and LC3. AST blocks JNK and reduces the levels of the autophagy-related proteins Beclin 1 and LC3. AST inhibits p38 and JNK and, consequently, it reduces autophagy by modulating MAPK-mediated release of Beclin 1. Dotted lines represent autophagy activation by astaxanthin (by inhibiting Akt activation) or p38 (by increasing Beclin 1 level).

Autophagy is the lysosomal degradation pathway that removes unused or damaged cellular components as well as toxic cytosolic materials. Basal autophagy promotes cell survival, but aberrant autophagy, which occurs under pathological conditions, is deleterious. The autophagy pathway provides the opportunity for the development of therapeutics for the prevention or treatment of a wide variety of diseases. Autophagy is regulated through several major signaling pathways that involve AMPK, PI3K/Akt, and MAPKs. The carotenoid astaxanthin prevents, via Akt and MAPK signaling, inflammatory damage and inhibits cellular death in neurodegeneration and inflammatory diseases. In cancer models, astaxanthin exhibits anti-proliferative effects through the signaling that regulates autophagy. In addition, several studies have demonstrated the potential of astaxanthin in regulating aberrant signaling and autophagy in disease. The signaling pathways, which regulate autophagy and autophagy-related proteins that are affected by astaxanthin, are displayed in Table 2 and Figure 1. The experimental findings that are covered in this review support the use of astaxanthin as a promising therapeutic agent in the treatment of diseases that are induced by the dysregulation of autophagy.

## Figures and Tables

**Table 1:** Signaling pathways that regulate autophagy.

	Experimental Model	Regulation of Signaling Mediators	Regulation of Autophagy	Ref.
AMPK	Oxygen deprivation of immortalized mouse embryo fibroblasts	AMPK activation Tuberous sclerosis complex 2 (TSC2) activation decreased phosphorylation of mTORC1 substrates	Autophagy ↑ (LC3 conversion, LC3 accumulation, autophagosome formation)	[35]
	Treatment of human breast epithelial cells with TRAIL	Transforming growth factor beta-activated kinase 1 (TAK1)-mediated AMPK activation	Autophagy ↑ (LC3II accumulation)	[36]
	Resveratrol treatment in chronic myelogenous leukemia cells	JNK activation c-jun phosphorylation AMPK activation decreased phosphorylation of mTOR and its substrates	Autophagy ↑ (p62 degradation, LC3II accumulation)	[37]
	Increased free cytosolic Ca^2+^ in MCF-7 breast cancer cells	AMPK activation	Autophagy ↑ (autophagosome formation)	[38]
	Treatment with AMPK activator AICAR	AMPK activation mTOR inhibition unc-51-like kinase (ULK1) activation	Autophagy ↑ (LC3II accumulation, p62 degradation	[39]
	Glucose starvation	AMPK activation ULK1 phosphorylation by AMPK ULK1 activation	Autophagy ↑	[40,41]
PI3K/Akt	Knockdown of Akt isoforms in cancer cell lines	Akt inhibition	Autophagy ↑ (acidic vesicular organelle accumulation, autophagosome formation)	[43]
	Starvation	PI3K/Akt inactivation mTOR inactivation	Autophagy ↑ (autophagosome formation, autolysosomal vesicle formation, Atg1 expression, Atg8 accumulation)	[45,46]
	Treatment of atherosclerotic vascular smooth muscle cells with insulin-like growth factor 1 (IGF-1)	Akt activation	Autophagy ↓ (no autophagic vacuoles)	[47]
	IL-13 stimulation in HT-29 cells	PI3K stimulation Akt activation	Autophagy ↓	[48]
	Ceramide treatment in HT-29 cells	inhibition of Akt activation	Autophagy ↑ (proteolysis, autophagic vacuole accumulation, increased Beclin 1 expression)	[49]
	Increased ceramide pool in breast cancer MCF-7 cells by tamoxifen treatment	inhibition of Akt activation	Autophagy ↑ (increased Beclin 1 expression)	[49]
	Resveratrol treatment in rat hearts exposed to chronic intermittent hypoxia	decreased PI3K expression decreased Akt activation decreased mTOR activation	Autophagy ↑ (increased LC3 expression, increased LC3II/LC3Ⅰ ratio, decreased p62 expression)	[50]
	Brain-derived neurotrophic factor (BDNF) treatment in hypoxic-ischemic brain injury model	decreased Akt activation decreased mTOR activation	Autophagy ↑ (LC3II conversion, LC3II aggregation)	[51]
	Oleanolic acid treatment in diabetic nephropathy model	decreased PI3K expression decreased Akt activation decreased mTOR activation	Autophagy ↑ (increased LC3Ⅰ and LC3II expression, decreased p62 expression)	[52]
	Treatment of malignant glioma cells with PI3K inhibitor LY294002 or Akt inhibitor UCN-01	inhibition of Akt activation decreased phosphorylation of mTOR substrates	Autophagy ↑ (autophagic vacuole formation, acidic vesicular organelle accumulation)	[53]
	Matrine treatment in acute myeloid leukemia cells	decreased Akt activation decreased mTOR activation decreased phosphorylation of mTOR substrates	Autophagy ↑ (p62 degradation, LC3II accumulation)	[54]
	Paeonol treatment in ovarian cancer cells	decreased Akt activation decreased mTOR activation decreased phosphorylation of mTOR substrates	Autophagy ↑ (increased LC3II expression, p62 degradation, autophagosome formation, LC3-labled autophagic vacuolation)	[55]
	Akt activation in HeLa cells in normal or starvation condition	Akt activation	Autophagy ↓ (Beclin 1 phosphorylation, inhibition of ClassⅢPI3K signaling)	[56]
	Exposure of human umbilical vein endothelial cells (HUVEC) to cooking oil fumes-derived particle matters	decreased PI3K activation decreased Akt activation decreased mTOR activation	Autophagy ↑ (autophagosome formation, increased LC3 puncta, increased Beclin 1 expression, increased LC3II/LC3Ⅰ ratio)	[57]
	High glucose exposure	decreased Akt activation decreased mTOR activation	Autophagy ↑ (increased LC3II expression, increased Beclin 1 expression, p62 degradation)	[58]
JNK	Treatment with neurotoxic N-methyl-d-aspartate (NMDA)	JNK activation increased c-jun phosphorylation increased c-fos expression	Autophagy ↑ (autophagic vacuole formation)	[59]
	Treatment with caspase inhibitor z-VAD	JNK activation Atg7 expression Beclin 1 expression	Autophagy ↑ (autophagic vacuole formation)	[60]
	Oncogenic H-ras infection	JNK-activating kinase, MAP kinase kinase 7 (MKK7) phosphorylation JNK activation increased Atg5 expression	Autophagy ↑ (acidic vacuole formation, LC3II accumulation, increased LC3 puncta, LC3 and LAMP-1 colocalization)	[61]
	Activation of CD4+ T cells	JNK signaling activation	Autophagy ↑ (increased LC3 puncta)	[62]
	Treatment of ceramide in cancer cells or neural progenitor cells	JNK activation increased c-jun phosphorylation	Autophagy ↑ (increased LC3 puncta, LC3II accumulation, autophagic vacuole formation, acidic vesicular organelle accumulation, increased expression of LC3, increased expression of Beclin 1)	[63,64]
	Ectopic expression of histone deacetylase 6 (HDAC6) in liver cancer cell lines	JNK activation increased c-jun phosphorylation	Autophagy ↑ (autophagic vacuole formation, LC3II accumulation, increased Beclin 1 expression)	[65]
	Exposure of human tumor cells to 1,3-dibutyl-2-thiooxo-imidazolidine-4,5-dione(C1)	JNK activation increased total and phosphorylated c-jun expression	Autophagy ↑ (increased LC3II expression, increased LC3II puncta, autophagosome formation, autophagic vacuole formation, Atg5-Atg7 conjugation)	[66]
	Bufalin treatment in human colon cancer cells	JNK2 activation	Autophagy ↑ (increased LC3 puncta, LC3II conversion, increased Atg5 expression, increased Beclin 1 expression)	[67]
	Nutrient starvation	JNK activation Multisite phosphorylation of Bcl-2 Beclin 1 dissociation	Autophagy ↑	[70]
	Ceramide treatment in cancer cell lines	JNK1 activation Multisite phosphorylation of Bcl-2 Beclin 1 dissociation	Autophagy ↑	[71]
	Human single-chain fragment variable, HW-1, treatment on cancer cells	JNK activation Beclin 1 expression	Autophagy ↑ (autophagic vesicle accumulation, increased LC3 puncta)	[72,73]
p38	LPS stimulation in microglia	p38α activation ULK1 phosphorylation ULK1-Atg13 complex disruption	Autophagy ↓ (decreased LC3II expression, increased p62 expression, decreased autophagosome number)	[74]
	TNF-α treatment in murine fibroblast L929 cells	decreased p38 activation decreased NF-κB	Autophagy ↓ (autophagic vacuole formation, LC3 puncta, LC3II expression, Beclin 1 expression)	[75]
	E Platinum treatment in gastric carcinoma cells	decreased Akt activation decreased p38 activation decreased mTOR activation	Autophagy ↑ (increased LC3 puncta, increased LC3II/LC3Ⅰratio, autolysosome formation, increased expression of lysosomal markers LAMP-1 and cathepsin D)	[76]
	Tumor treatment with Ganoderma Lucidum triterpenes	p38α signaling activation	Autophagy ↑ (autophagic vacuole formation, increased LC3 expression, increased Beclin 1 expression)	[77]
	Treatment of colon cancer HCT116 cells with 5-fluorouracil	p38 activation	Autophagy ↓	[79]
	Treatment of HCT116 cells with active metabolite of irinotecan	MAPK14/P38α kinase activation	Autophagy ↑ (increased LC3II expression, autophagic vacuole formation, punctated LC3)	[80]
	Oridonin treatment in human cervical carcinoma HeLa cells	Increased p38 expression Increased JNK expression Increased p38 phosphorylation Increased JNK phosphorylation	Autophagy ↑ (autophagic vacuole formation, increased LC3II expression, increased Beclin 1 expression)	[81]
	LPS stimulation in skeletal muscle	p38 activation	Autophagy ↑ (expression of Beclin 1, Atg7, Atg12)	[82]
	H_2_O_2_ stimulation in myotubes	p38 activation	Autophagy ↑ (increased expression of Atg7)	[82]
	Resveratrol treatment in H_2_O_2_-stimulated embryonic rat heart-derived cells	p38 activation	Autophagy ↑ (autophagosome formation, increased LC3II expression, increased Beclin 1 expression)	[83]
	ER stress induced by pharmarcologic agents	p38 activation	Autophagy ↑ (autophagic vacuole formation, autophagosome formation, increased Beclin 1 expression)	[84]
	Transfection of NIH/3T3 fibroblasts with Gadd45β	p38 activation	Inhibition of the autophagic flux (increased LC3 puncta, decreased autolysosomal degradation, Atg5 phosphorylation)	[85]
	Activation of p38 in starved cells	p38α kinase activation loss of membrane bound p38IP inhibition of p38IP – mAtg9 interaction	Autophagy ↓ (autophagosome formation, increased LC3II expression, p62 degradation)	[86]
	Selenite treatment on colon cancer cells	p38 activation JNK activation	Autophagy ↑ (autophagic vacuole formation, p62 degradation, increased Beclin 1, Lamp-1 and cathepsin D expression, LC3II conversion)	[87]

**Table 2:** The effect of astaxanthin on the signaling pathways that impact autophagy.

Experimental Model	Astaxanthin Dose	Regulation of Signaling Mediators	Notable Results	Ref.
Oleic acid-induced hepatic steatosis	10 μM	increased p-AMPK/AMPK ratio	decreased cell death reduced cell damage	[88]
Performance endurance test	0.02% esterified astaxanthin from *Haematococcus pluvialis*	increased total AMPK expression	increased endurance performance	[89]
H_2_O_2_-stimulation in mouse neural progenitor cells	10 ng/mL	increased p-Akt expression decreased p-p38 expression	decreased cell death reduced cytotoxicity increased cell proliferation	[90]
Acetaldehyde-induced neurotoxicity	50 ng/mL	increased p-Akt expression decreased p-p38 expression	decreased cell death reduced cytotoxicity	[91]
Glutamate-induced cytotoxicity in neurotoxicity	5 μM	increased p-Akt expression activation Nrf2	decreased cell death reduced cytotoxicity	[92]
Homocysteine-induced neurotoxicity	5 μM	increased p-Akt expression	decreased cell death reduced cytotoxicity	[93]
H_2_O_2_ stimulated retinal pigment epithelial cells	20 μM	increased p-Akt expression activation of Nrf2	decreased cell death	[94]
Isoflurane-induced neurotoxicity	8 μM 100 mg/kg	increased p-Akt/Akt ratio	decreased cell death reduced cytotoxicity	[95]
Chronic organophosphorus pesticide exposure	50 mg/kg/d	increased p-PI3K expression increased p-Akt expression	reduced cytotoxicity	[96]
Pilocarpine-induced status epilepticus	30 mg/kg	increased p-Akt/Akt ratio	decreased cell death reduced cytotoxicity	[97]
Spinal cord injury	10 μL of 0.2 mM	increased expression of p-Akt	decreased cell death	[98]
Neural progenitor/stem cells	10 ng/mL	activation of PI3K increased expression of p-Akt	increased cell proliferation	[99,100]
Hypoxia and reoxygenation-stimulated Kupffer cells	10 μM	increased p-Akt expression increased mTOR expression	decreased cell death	[101]
Hypoxia and reoxygenation-induced ischemia-reperfusion injury	10 μM	decreased p-JNK expression decreased p-p38 expression	Decreased cell death	[101]
Cognitive deficit in diabetic rats	10, 20, 40 mg/kg	increased PI3K expression increased Akt expression	decreased oxidative cell death	[102]
Cognitive deficit in diabetic rats	50, 100 mg/kg	increased total Akt and p-Akt expression	decreased oxidative cell death	[103]
Early acute kidney injury	20 mg/kg	increased p-Akt expression	decreased cell death	[104]
Early burn wound	5, 10, 20 mg/kg	increased p-Akt expression	decreased cell death	[105]
Brain injury post-subarachnoid hemorrhage	20 μL of 0.1mM	increased p-Akt expression	decreased cell death	[106]
Human hepatocellular carcinoma cells	100, 200, 300 μM	decreased p-Akt/Akt ratio inhibition of NF-κB inhibition of Wnt/β-catenin	inhibition of cell proliferation loss of cell viability	[107]
Hamster model of DMBA-induced oral cancer	15 mg/kg BW	decreased total Akt and p-Akt expression inhibition of NF-κB inhibition of Wnt/β-catenin	inhibition of cell proliferation loss of cell viability	[108]
Oral squamous cell carcinoma	400 μM 15 mg/kg	inhibition of PI3K decreased p-Akt expression inhibition of NF-κB/STAT3	loss of cell viability enhanced cytotoxicity	[109]
Human non-small cell lung cancer cells	20 μM	inactivation of Akt kinase	loss of cell viability enhanced cytotoxicity	[110]
High fat diet-induced nonalcoholic fatty liver disease model	30 mg/kg	decreased p-Akt/Akt ratio decreased p-GSK3 expression	induction of hepatic autophagy	[111]
Cerulein-induced acute pancreatitis	20, 40 mg/kg	increased p-STAT3 expression increased expression of Bcl-2 decreased expression Beclin 1	inhibition of cerulein-induced autophagy	[112]
Bile duct ligation-induced liver fibrosis	40, 80 mg/kg	decreased NF-κB expression and activation	Inhibition of hepatic autophagy	[113]
UVB irradiated human lens epithelial cells	2 μmol/L	decreased JNK1 and JNK2 activation decreased p-p38 expression	alleviated oxidative damage	[114]
Dextran sulphate sodium-induced colitis TNF-α stimulation in colonic epithelial cell	0.02, 0.04%	decreased p-JNK expression decreased p-p38 expression	alleviated inflammation	[115]
Cobalt-induced cytotoxicity	1, 10, 20 nM	decreased p-JNK expression decreased p38 expression decreased Akt expression	reduced cytotoxicity	[116]
Insulin signaling in skeletal muscle	5, 10, 20 μM	increased p-Akt expression decreased p-JNK expression	increased glucose uptake	[117]
LPS-stimulated inflammation	50, 100, 200 mg/kg	decreased p-JNK expression decreased p-p38 expression	alleviated inflammation	[118]
Palmitate stimulation in mesenchymal stem cells	10 μM	decreased p-JNK expression decreased p-p38 expression	decreased cell death reduced cytotoxicity	[119]
Acetaminophen-induced liver injury	30, 60 mg/kg/d	decreased total JNK and p-JNK expression decreased p-p38 expression	decreased cell death reduced cytotoxicity	[120]
High fructose and high fat diet-fed mice	2 mg/kg	increased total JNK and p-JNK expression	alleviated inflammation	[121]
Palmitate-induced cytotoxicity	10 μmol/L	decreased p-JNK expression decreased p-Akt expression	alleviated inflammation	[122]
Fluctuating high glucose exposure in human vascular endothelial cells	0.05, 0.1, 0.5 μM	decreased p-JNK expression decreased p-p38 expression	decreased cell death reduced cytotoxicity	[123]
Human colon cancer cells	15, 25 μg/mL *Haematococcus pluvialis* extract	increased p-JNK expression increased p-p38 expression decreased p-Akt expression	inhibition of cell proliferation loss of cell viability	[124]
ConA-induced autoimmune hepatitis	20, 40 mg/kg	decreased p-JNK expression	Inhibition of hepatic autophagy	[125]
Human lung carcinoma cells	20 μM	increased p-p38 expression	loss of cell viability enhanced cytotoxicity	[126]
6-hydroxydopamine-induced neurotoxicity	20 μM	decreased p-p38 expression	decreased cell death reduced cytotoxicity	[127]
Beta-amyloid-induced neurotoxicity	5, 10 μM	decreased p-p38 expression	decreased cell death reduced cytotoxicity	[128]
Glutamate-induced neurotoxicity	50 μg/L	decreased p-p38/p38 ratio	decreased cell death reduced cytotoxicity	[129]
β-amyloid peptide-induced neurotoxicity	0.1 μM	decreased p-p38 expression	decreased cell death reduced cytotoxicity	[130]
Environmental tobacco smoke-induced cognitive deficits	40, 80 mg/kg	decreased p-p38 expression	reduced cytotoxicity	[131]
Cyclophosphamide-induced hepatocarcinogenesis	25 mg/kg	decreased p-p38 expression	inhibition of early hepatocarciongenesis	[132]
Spinal cord injury	10 μL of 0.2 mM	decreased p-p38 expression	alleviated neuropathy	[133]
IL-1β-induced osteoarthritis in chondrocytes	10, 50 μM	decreased p-p38 expression	lower MMP level	[134]
Hepatic ischemia reperfusion injury	60 mg/kg	decreased p-JNK expression decreased p-p38 expression decreased p-ERK expression	inhibition of hepatic autophagy	[135]
